# Yunnan Baiyao Ameliorates Rheumatoid Arthritis in Rats by Shifting the Th17/Treg Cell Balance and Preventing Osteoclast Differentiation

**DOI:** 10.1155/2022/3764444

**Published:** 2022-02-07

**Authors:** Xiaobin Ren, Mingzhu Zhang, Wanli Zhang, Jing Xie, Hongcan Luo, Hongming Zhang, Hongbing He

**Affiliations:** Department of Periodontology, The Affiliated Stomatology Hospital of Kunming Medical University, Kunming, Yunnan 650031, China

## Abstract

Yunnan Baiyao (YNB) is a traditional Chinese medicine that possesses anti-inflammatory effects. Previously, we have demonstrated the effects of YNB in rheumatoid arthritis (RA) animal models; however, the underlying mechanisms are unclear. In the present study, we aimed to investigate the effects of YNB on the T-helper (Th)17/T-regulatory (Treg) cell balance in a collagen-induced arthritis rat model orally administrated YNB or methotrexate, a widely used therapeutic agent for treating RA. Our results showed that YNB treatment significantly decreased the voix pedis thickness and joint functionality scores and alleviated joint histopathology in these rats. These YNB-induced effects were achieved by decreasing the number of Th17 cells and increasing that of Treg cells in the spleen. Moreover, the interleukin- (IL-) 17 level considerably decreased in the serum of YNB-treated rats, whereas the IL-10 level significantly increased. Furthermore, YNB could inhibit RANKL-induced osteoclast formation by regulating the tumor necrosis factor receptor-associated factor 6/NF-*κ*B/nuclear factor of the activated T-cell pathway. In summary, our study shows that YNB exhibits antiarthritic activity by decreasing the ratio of Th17/Treg cells, regulating the cytokine balance, and inhibiting osteoclast activation, providing an experimental basis that supports the use of this traditional Chinese medicine for the clinical treatment of RA.

## 1. Introduction

Rheumatoid arthritis (RA) is a systemic heterogeneous autoimmune disease of unknown etiology mainly manifested by chronic inflammatory polyarthritis [1]. It is characterised by T-lymphocyte infiltration-based chronic synovitis that eventually leads to arthritis and bone destruction [2]. In recent years, with the discovery of T-helper (Th) cells in the CD4^+^ T-cell compartment, the roles of these cells in RA have been unveiled. In particular, the proinflammatory effects of Th17 cells, anti-inflammatory effects of regulatory T (Treg) cells, and balance of the Th17/Treg cell ratio in RA have been the focus of several studies. Moreover, as a target for the treatment of RA, the balance of Th17/Treg cells has become a hotspot of research on the pathogenesis of RA [3].

Currently, therapeutic drugs for RA mainly consist of immunosuppressive agents, hormones, and biological agents, with clear curative effects, but the frequency and severity of their adverse effects and economic burden are still obstacles that need to be overcome in clinical practice [4]. Conversely, traditional Chinese medicine has a long history in the treatment of RA, for which diverse treatment methods have been used. These methods are simple, effective, and cheap and rely on multiple components, which allows tackling multiple pathways and targets [5]. At present, many kinds of traditional Chinese medicines have been proven to be effective in treating arthritis [6]. Yunnan Baiyao (YNB) is a widely used Chinese herbal medicine mainly composed of Radix Notoginseng, Forest Musk, Borneolum Synthcticum, Rhizoma Paridis (Chonglou), and Radix Aconiti Kusnezoffii [7]; it exerts a good therapeutic effect on bleeding, ulcers, infections, hemangiosarcoma, and inflammatory bowel disease [8–11]. Previous studies have also shown that the immunosuppressive effect of YNB is attributable to its highly selective cytotoxicity on B and T lymphocytes as well as the inhibition of tumor necrosis factor- (TNF-) *α* and interferon expression [12]. Our previous studies have shown that YNB can inhibit joint inflammation and bone destruction in a rat arthritis model [7, 13]. However, the immunological mechanism underlying the effects of YNB in the treatment of RA is still not clear.

As the ratio of Th17/Treg cells is crucial for the pathogenesis of RA, we hypothesised that the antiarthritic effects of YNB may be mediated via the regulation of the Th17/Treg cell balance. Therefore, in the present study, we focused on the potential anti-RA mechanisms induced by YNB, comparing them with those induced by methotrexate (MTX), one of the most frequently prescribed medicines for treating RA, to provide an experimental basis for the clinical treatment of this disease.

## 2. Materials and Methods

### 2.1. Reagents

YNB was obtained from Yunnan Baiyao Group Co., Ltd. (Kunming, China). MTX was purchased from Sine Pharmaceutical Co., Ltd. (China). Freund's complete adjuvant (FCA) and bovine type II collagen (CII) were purchased from Sigma-Aldrich (St. Louis, MO, USA). Aseptic 0.1 mol/L acetate solution containing 2 mg/mL C-II (bovine type II collagen) was settled at 4°C overnight and then mixed with the same volume of FCA. The fresh mixture (containing C-II diluted in acetate solution and FCA) was used as the model construction reagent (MCR). The tartrate resistant acid phosphatase (TRAP) staining kit was acquired from Sigma-Aldrich. The antinuclear factor of activated T cells 1 (NFATc1), anti-TNF receptor-associated factor 6 (TRAF6), anti-NF-*κ*Bp65, and antiphosphorylated (phospho)-NF-*κ*Bp65 antibodies were purchased from Cell Signaling Technology (Beverly, MA, USA); antiosteoprotegerin, anti-RANKL, and anti-beta actin antibody were purchased from Abcam (Shanghai).

### 2.2. RA Model Construction and Treatment

Specific-pathogen-free male Sprague Dawley rats, aged 7-8 weeks and weighing 180–220 g, were provided by the Experiment Animal Center of the Kunming Medical University (KMU). Animal experiments were carried out under the monitoring of the Experiment Ethical Committee of the KMU. In total, 80 rats were used in the study; they were randomly divided into four groups: control (group C), model (group R), YNB (group Y), and MTX (group M). Group R, group Y, and group M were treated with 0.2 mL MCR by intracutaneous injection on multiple sites of the root of the tail on day 1. At the same time, injection of 0.1 mL MCR in the left rear voix pedis was carried out for each rat. RA symptoms appeared within 7 days. On days 7 and 21, only 0.2 mL MCR injection was repeated on the tail of each rat. [7]. The RA model rats in group R received only solvent without YNB throughout the experimental period. From days 7 to 35, rats in the other three groups were treated differently: group Y received 1 mL of YNB (50 mg/mL aseptic aqueous suspension) intragastrically once every day; group C was treated with the same volume of physiological saline; and group M received 1 mL of MTX (0.2 mg/mL) intragastrically once every week [14]. In each group, 10 rats were anaesthetised with ether on weeks 2 and 4, and the relevant pathological parameters were determined as necessary. Blood was collected from the abdominal aorta and centrifuged to obtain the serum, which was immediately stored at −70°C for enzyme-linked immunosorbent assay (ELISA). During the experimental period, all rats had free access to food and water, and the lighting duration was 12 h every day. The rats were sacrificed by cervical dislocation after blood collection, and all efforts were made to minimise suffering to them.

### 2.3. Histopathological Analysis

Joint samples were collected and fixed in 4% paraformaldehyde fixation solution for 24 h. Subsequently, joint samples were dehydrated, embedded in paraffin, and then, sectioned using a microtome (5 *μ*m). The sections were stained with hematoxylin and eosin to evaluate histopathological changes.

### 2.4. Evaluation of Arthritis

Arthritis severity was evaluated by examining changes in the voix pedis thickness and joint functionality scores. Joint functionality scores were evaluated by independent physicians according to the following criteria: 0, no appreciable symptoms; 1, discernible red spot(s) or slight swell; 2, intermediate swell; 3, serious swell; and 4, deformed joint(s) with stiffness. The highest total score of the four limbs was 16 for each rat [7].

### 2.5. RT-PCR

Total RNA was isolated from the joint samples using TRIzol reagent (Invitrogen; Thermo Fisher Scientific, Inc., Waltham, MA, USA), according to the manufacturer's protocol. The RT reactions were performed using the PrimeScript™ 1st Strand cDNA Synthesis Kit (Takara Biotechnology Co., Ltd. Dalian, China). An equal amount of total RNA (2 *μ*g) was used for cDNA synthesis, according to the manufacturer's protocol. The RT reaction products were stored at −20°C. Primers used for PCR were designed by Shanghai Sangon Biotechnology Co., Ltd. (Shanghai, China) and are presented in Table 1. The PCR amplification was carried out using gene-specific PCR primers, 2 *μ*L of cDNA, 12.5 *μ*L of GoTaq® Green Master Mix 2X (Promega Corporation, Madison, WI, USA), and 8.5 µL of ddH_2_0. The reaction conditions for RANKL, OPG, and GAPDH PCR were 30 cycles of denaturation at 94°C for 45 s, annealing at 59°C for 45 s, and extension at 72°C for 7 min. The PCR products were run on a 1.5% agarose gel and stained with ethidium bromide. The band intensity was determined using Quantity One software v4.6.2 (Bio-Rad Laboratories, Inc., Hercules, CA, USA). The results are presented as the ratio of target mRNA to *ß*-actin mRNA.

### 2.6. Western Blotting

Total protein was extracted from cultured RAW264.7 cells using the RIPA Lysis and Extraction Buffer (Boster Biotechnology, Boster, Wuhan, China). Proteins were separated by sodium dodecyl sulphate polyacrylamide gel electrophoresis (10%) and then transferred onto polyvinylidene fluoride membranes (Millipore, Billerica, MA, USA). The membranes were blocked with 5% BSA in Tris‐buffered saline with Tween 20 (TBST) for 60 min and then incubated overnight at 4°C with the following primary antibodies: anti-NF‐*κ*Bp65, anti-phospho‐NF‐*κ*Bp65, anti-TRAF6, anti-NFATc1, antiosteoprotegerin (OPG), anti-RANKL, and anti-beta actin. The membranes were washed three times with TBST and incubated with HRP‐conjugated secondary antibodies for 1 h. Finally, the proteins were detected using electrochemiluminescence reagents (Thermo Fisher Scientific), and the intensity of bands was quantified using Image Lab 5.1 software (Bio‐Rad, Hercules, CA, USA) and normalised to that of the control.

### 2.7. Enzyme-Linked Immunosorbent Assay

Blood was centrifuged for 10 min to collect plasma. ELISA kits (Sigma-Aldrich; Merck Millipore) were used to detect plasma levels of interleukin- (IL-) 10 and IL-17, according to the manufacturer's instructions.

### 2.8. Isolation of Spleen Lymphocytes

Sprague Dawley rats were sacrificed and immersed in 75% alcohol for 1-2 min. Thereafter, 4-5 mL of EZSepTM mouse 1 × lymphocyte separation solution (shaken before use) was added into a 60 mm culture dish, and a nylon mesh (200 mesh sieve) was fixed with a pair of tweezers. The spleens were extracted, cut using a pair of scissors, placed on the nylon mesh, and gently ground with a syringe plunger, to ensure that the dispersed single cells could enter the lymphocyte separation solution. The spleen cell suspension was immediately transferred into a centrifuge tube, which was filled with approximately 200 *μ*L of RPMI1640 medium before centrifugation. After centrifugation at 800 × *g* for 30 min, the lymphocyte layer was aspirated, and 10 mL of RPMI1640 medium at 1500 rpm was added. After centrifuging again for 10 min, the supernatant was decanted, and cells were resuspended in 3–5 mL of Lympho-SpotTM serum-free medium and counted.

### 2.9. Flow Cytometric Analysis

Rat spleen lymphocytes were washed with PBS to adjust the cell concentration to 1 × 10^7^ cells/mL. Fifty microliters of spleen lymphocyte suspension was placed in a Falcon tube, and fluorescein isothiocyanate- (FITC-) labelled anti-mouse/rat IL-17A and mouse FITC-labelled anti-rat CD4 and PE-labelled anti-CD25 monoclonal antibodies were added, and the solution was incubated for 20 min at room temperature, in the dark. A blank control group (without antibodies) was also prepared. Cells were resuspended in 500 *μ*L of PBS, and then, the flow cytometer Epics XL (Beckman Coulter, USA) was used to detect changes in the Th17/Treg cell ratio.

### 2.10. Cell Culture and *In Vitro* Osteoclastogenesis Assay

The murine macrophage cell line RAW264.7 was purchased from Beijing Beina Chuanglian Biotechnology Research Institute (BNCC100584) and cultured in *α* minimum essential medium (Thermo Fisher Scientific, HyClone, Beijing, China) supplemented with 10% fetal bovine serum (HyClone, USA) and antibiotics (120 U/mL penicillin and 75 *μ*g/mL streptomycin; Invitrogen, USA) in a 5% CO_2_ atmosphere at 37°C. RAW264.7 cells were incubated in a medium containing receptor activator of nuclear factor kappa-B ligand (RANKL) (100 ng/mL) [15] with or without YNB (5, 10, or 20 *μ*g/mL) [7] for 7 days to induce osteoclastogenesis. After this period, the cells were fixed in 4% paraformaldehyde for 15 min. After washing with phosphate‐buffered saline (PBS), the cells were incubated for 60 min at 37°C in the dark with a mixture of TRAPs, according to the manufacturer's instructions. TRAP^+^ cells with ≥3 nuclei were defined as osteoclasts. Total RNA and protein were collected for reverse transcription (RT) polymerase chain reaction (PCR) and western blotting, respectively.

### 2.11. Statistical Analysis

The experimental data were analysed using SPSS version 17.0 (SPSS Inc., Chicago, IL, USA). All data are presented as mean ± standard deviation; the data were analysed using the analysis of variance followed by Dunnett's test. Differences with *p* < 0.05 were considered statistically significant.

## 3. Results

### 3.1. YNB Improves Clinical Symptoms in Collagen-Induced Arthritis (CIA) Rats

**Figure 1:**
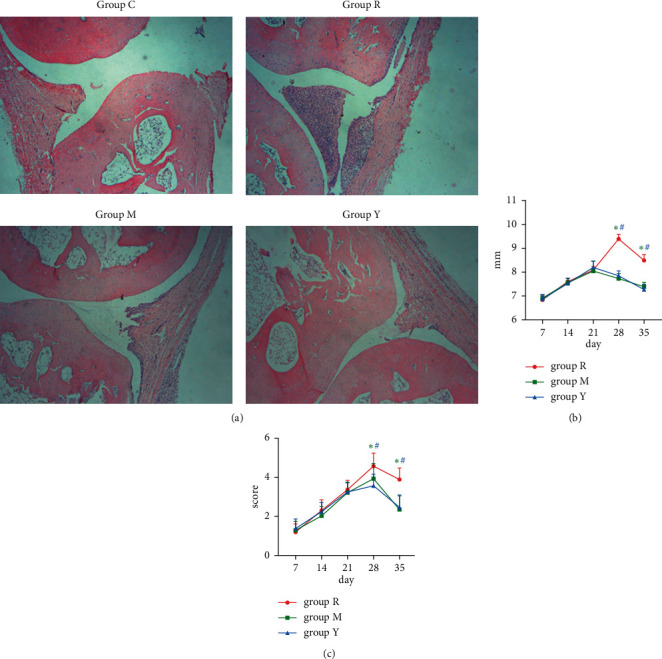
Effects of YNB on CIA rats. (a) Representative images of joint histopathology analysis with hematoxylin and eosin staining (magnification ×40). (b) Voix pedis thickness and (c) joint functionality scores were monitored every 7 days after the second immunisation.

The effects of YNB were evaluated using the CIA model. The voix pedis thickness and joint functionality scores were determined every week after the second immunisation. Before day 21, there were no significant differences in the voix pedis thickness and joint functionality scores among the three groups. However, on day 28, they reached the maximum in the group, after which the arthritis score gradually decreased. On day 35, after the oral administration of YNB and MTX, they considerably decreased (*p* < 0.05) (Figure 1(b) and 1(c)). To understand the actual disease state, digiti pedis joint samples were collected on day 28. Histological analysis showed that the structure of the normal joint was complete in group C, and there were no signs of inflammation. However, inflammatory cell infiltration and synovium hyperplasia were observed in the joints of group R, and these symptoms were alleviated by the administration of both MTX and YNB (Figure 1(a)).

### 3.2. YNB Regulates the Th17/Treg Cell Balance in the Spleens of CIA Rats

**Figure 2:**
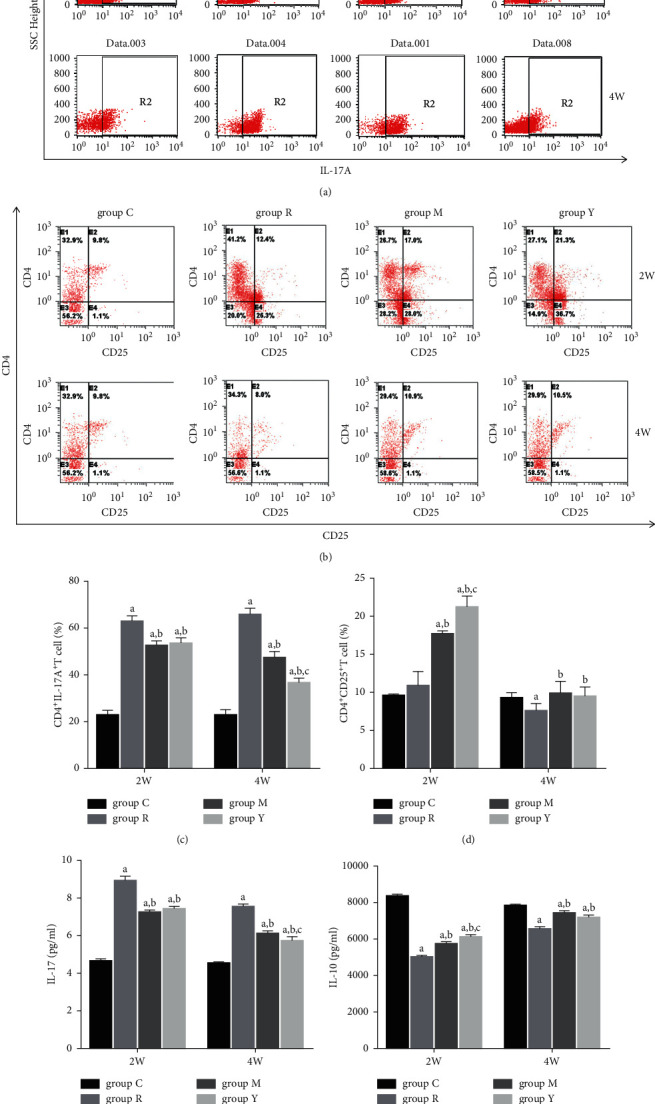
Effects of YNB on the ratio of Th17/Treg cells and inflammation-related cytokines in CIA rats. Splenocytes were obtained at weeks 2 and 4 after intragastric treatment, and the number of CD4^+^IL-17A^+^ Th17 cells (a and c) and CD4^+^CD25^+^ Treg cells (b and d) was analysed by flow cytometry. The levels of IL-17 (e) and IL-10 (f) in plasma were measured by ELISA. Data are presented as mean ± SD. ^a^*p* < 0.05 vs. group C; ^b^*p* < 0.05 vs. group R; and ^c^*p* < 0.05 vs. group M.

To determine whether YNB contributes to the immune balance in RA, the Th17/Treg cell compartment in the spleen was investigated via flow cytometry after the oral administration of the treatments. Both YNB and MTX treatments decreased the percentage of Th17 cells (Figures 2(a)–2(c)) and increased the percentage of Treg cells (Figures 2(b)–2(d)) in splenocytes compared with those in group R.

### 3.3. YNB Regulates the Level of Cytokines in the Plasma of AA Rats

To analyze the effects of YNB on inflammation-related cytokines in the plasma, the levels of IL-17 and IL-10 were measured by ELISA. The level of IL-17 in AA rats was higher than that in group C, whereas the level of IL-10 showed the opposite trend. After treatment with YNB and MTX, the level of IL-17 decreased, whereas that of IL-10 significantly increased (Figures 2(e) and 2(f)). These results suggest that the ability of YNB to induce protection against AA involves the regulation of the balance between proinflammatory cytokines and anti-inflammatory cytokines.

### 3.4. YNB Regulates the Expression of RANKL and OPG in the Joint Tissue

**Figure 3:**
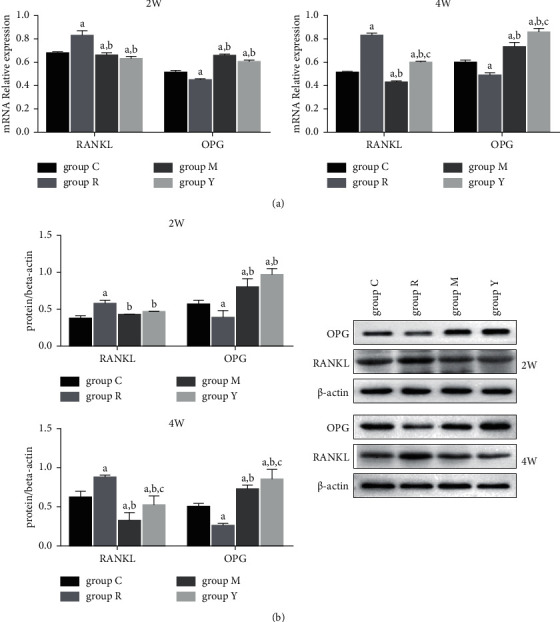
Effects of YNB on the expression of RANKL and OPG in the joint tissue of CIA rats. (a) RT-qPCR analyzes the expression of OPG and RANKL mRNA. (b) Western blotting analyzes the expression of OPG and RANKL. Data are presented as mean ± SD. ^a^*p* < 0.05 vs. group C; ^b^*p* < 0.05 vs. group R; and ^c^*p* < 0.05 vs. group M.

As our previous study showed that YNB can inhibit the formation of osteoclasts in inflamed joints [7], we analyzed the expression of RANKL and osteoprotegerin (OPG) in this tissue. The RT-PCR results showed that the levels of RANKL and OPG mRNA were significantly higher and lower, respectively, in group R than in group C (Figure 3(a)) and were downregulated and upregulated in groups M and in groups Y. Furthermore, the Western blotting showed the same results (Figure 3(b)).

### 3.5. YNB Inhibits RANKL-Induced Osteoclastogenesis

**Figure 4:**
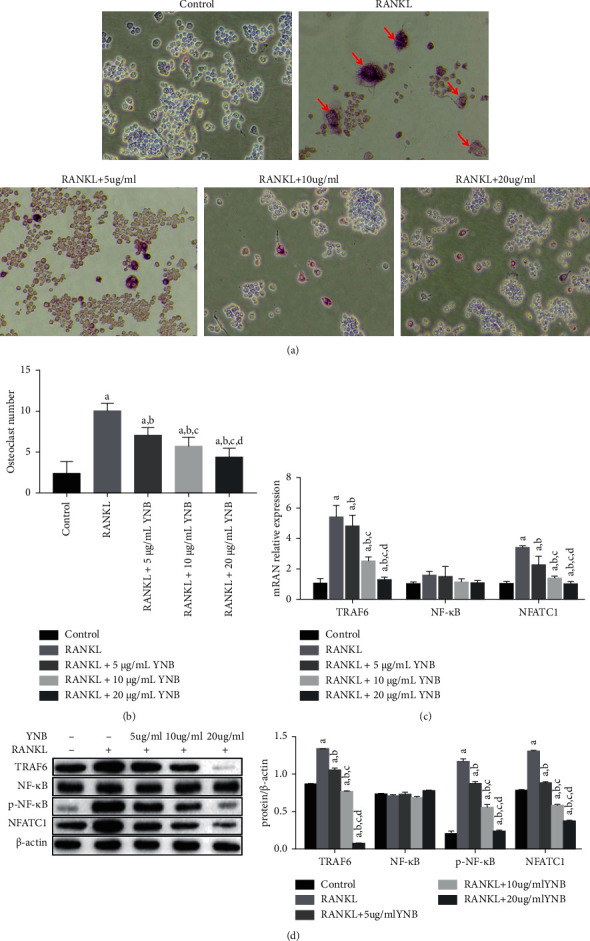
Effects of YNB on RANKL‐induced osteoclastogenesis. (a, b) RAW264.7 cells induced by RANKL (100 ng/mL) were treated with YNB (5, 10, or 20 *μ*g/mL) for 7 days, fixed, and stained with a TRAP staining kit to detect TRAP^+^ cells (magnification ×200). (c) RT‐PCR analysis of TRAF6, NF-*κ*B, and NFATC1 mRNA. (d) Protein levels of phospho-NF-*κ*B, NF-*κ*B, TRAF6, and NFATC1 were evaluated by western blotting. Data are presented as mean ± SD. ^a^*p* < 0.05 vs. control; ^b^*p* < 0.05 vs. RANKL; ^c^*p* < 0.05 vs. RANKL +5 *μ*g/mL; and ^d^*p* < 0.05 vs. RANKL +10 *μ*g/mL.

RANKL is a key regulator of osteoclast activation. As our results showed that YNB can reduce the expression of RANKL in inflamed joints, we investigated the protective effect of YNB against bone destruction in inflamed joints. To this end, we established an RANKL-induced RAW264.7 osteoclast differentiation model and administered YNB. The results showed that YNB inhibited osteoclast differentiation (Figures 4(a) and 4(b)). As the TRAF6/NF-*κ*B/NFATc1 pathway is a critical signalling pathway associated with RANKL-mediated osteoclastogenesis, we conducted further RT-PCR and western blotting analyses. We observed that the levels of TRAF6 and NFATc1 mRNA significantly increased after incubation with RANKL. However, the mRNA and protein levels of TRAF6 and NFATc1 decreased in a dose-dependent manner after treatment with YNB (Figures 4(c) and 4(d)). Regarding NF-*κ*B, YNB had no effect on the total protein level, but promoted its phosphorylation. These results indicate that YNB suppresses osteoclastogenesis in a dose-dependent manner, contributing to the inhibition of articular bone destruction.

## 4. Discussion

RA is a chronic autoimmune disease, and its development involves a variety of immune cells, such as dendritic cells, T cells, and B cells. The main pathological manifestations of RA include synovial hyperplasia and joint cartilage and bone destruction [16]. Studies have shown that the imbalance of the Th17/Treg cell ratio plays an important role in the pathogenesis of RA [17]. IL-17 secreted by Th17 cells and other proinflammatory factors promote joint inflammation and inhibit Treg cell differentiation. In turn, Treg cells produce IL-10, which can limit tissue damage caused by inflammatory reactions. The deficiency of IL-10 can stimulate Th1 and Th17 cell differentiation and inhibit that of Treg cells, leading to the occurrence of RA [18, 19]. In addition, it has been reported that Th17 cells promote bone erosion and destruction in a CIA model by regulating RANKL, which in turn mediates the formation of osteoclasts [20]. Therefore, restoring the Th17/Treg cell balance and inhibiting proinflammatory cytokines may control this inflammatory process and inhibit the destruction of joint bones.

The CIA model is the most commonly studied animal model of RA [21] and is usually used to study the molecular mechanisms underlying RA and the potential therapeutic effects of different interventions. As a traditional Chinese medicine compound, YNB has been used to treat various diseases, including bleeding, ulcers, infections, angiosarcoma, and inflammatory bowel disease. It has been reported that YNB exerts anti-inflammatory effects in RA rat models [7]. The present study demonstrated that YNB can also reduce paw swelling and the arthritis index. Moreover, our histological evaluation showed that YNB treatment can inhibit synovial hyperplasia and inflammatory cell infiltration, confirming that this traditional medicine exerts therapeutic and protective effects in RA rat models.

The Th17/Treg cell imbalance not only plays a key role in RA but also plays an important role in systemic lupus erythematosus (SLE) and uveitis. The changes in cytokines secreted by Th17 and Treg cells are closely correlated with the pathogenesis of SLE and uveitis [22, 23]. Therefore, we isolated spleen lymphocytes from different groups of RA rats and analyzed the Th17/Treg cell ratio by flow cytometry. Our results showed that the number of Treg cells in the model group decreased and that of Th17 cells increased; the YNB treatment could reverse this trend. Simultaneously, we found that YNB increased the plasma IL-10 level and reduced the IL-17 level. These results indicate that YNB can reduce inflammation in the CIA model by regulating the balance of Th17/Treg cells. The drugs traditionally used for the treatment of RA are immunosuppressive agents, such as MTX. This study also showed that MTX can regulate the Th17/Treg cell ratio.

Joint bone destruction in RA is mainly mediated by osteoclasts. Osteoclasts are members of the monocyte/macrophage lineage and are the only cells involved in the bone resorption process of bone metabolism [24]. Studies have shown that the RANK/RANKL/OPG signaling pathway is important during osteoclast differentiation; the activation of osteoclasts depends on the binding of RANKL to its receptor RANK that is expressed in osteoclasts. As a soluble inducer of RANKL, OPG can block the RANK-RANKL interaction, inhibiting the formation of osteoclasts [25]. Studies have shown that Th17 cells, especially those producing IL-17, are involved in inflammatory bone destruction in RA and that RANKL expressed on the surface of Th17 cells can interact with RANK on precursors of osteoclasts, leading to osteoclast differentiation [26]. Furthermore, high local levels of IL-17 can significantly upregulate the expression of RANKL and its receptor RANK, enhance osteoclast activity, and then, disrupt bone metabolism, affecting the synovial fluid and aggravating bone destruction [27]. Therefore, in this study, we analysed RANKL and OPG mRNA levels in joint tissues and found that, in the model group, the former was significantly increased, whereas the latter was significantly reduced. Moreover, both YNB and MTX were able to downregulate RANKL and upregulate OPG. Western blotting also showed the same results. These results further prove that YNB can inhibit the formation of osteoclasts in inflamed joints, thereby preventing the destruction of joint bones, as shown in our previous research [7].

RANKL produced by Th17 cells is an important factor that mediates the activation of osteoclasts in local joint tissues. The present study shows that YNB can significantly inhibit RANKL expression, thereby inhibiting osteoclast formation and joint bone destruction. However, it is unclear if this effect is achieved only by downregulating RANKL expression or also by affecting RANKL-induced osteoclast activation. Studies have shown that RANKL is the main molecule that promotes osteoclast differentiation, while NFATc1 induces osteoclast maturation. The RANKL-RANK interaction promotes the binding of the intracellular region of RANK and TRAF6, activating downstream NF-*κ*B, among others, followed by the activation of NFATc1. In turn, the latter induces the expression of many osteoclast-specific genes (e.g., cathepsin K, tartrate-resistant acid phosphatase, and calcitonin receptor) to promote the differentiation of osteoclasts. Simultaneously, NFATc1 can also bind to the NFAT binding site on its precursor, promoting its own enlargement and, thereby, maintaining the continuous transmission of signals [28]. Therefore, we further analyzed the effect of YNB on RANKL-induced differentiation of RAW264.7 cells into osteoclasts in vitro and found that YNB could significantly reduce the formation of multinucleated osteoclasts. Moreover, western blotting and RT-PCR analyses showed that YNB reduced the expression of TRAF6 and NFATc1 induced by RANKL and that it had no effect on the expression of NF-*κ*Bp65, but induced its phosphorylation. Therefore, our results indicate that YNB can not only downregulate RANKL expression but also inhibit RANKL-induced osteoclast activation.

## 5. Conclusions

This study indicates that YNB can inhibit inflammation in a CIA model by regulating the balance of Th17/Treg cells, which results in the prevention of joint synovial hyperplasia. Furthermore, YNB can inhibit joint bone destruction by downregulating RANKL expression and preventing the formation of osteoclasts in joints. Therefore, this study provides a theoretical basis for the clinical application of YNB.

## Figures and Tables

**Table 1:** Primer sequences for RT quantitative PCR.

Gene	Sequence (5′-3′)		Product length (base pairs)
	Forward primer	Reverse primer	
*c-Fos*	GAGAATCCGAAGGGAACGGAAT	GCAACGCAGACTTCTCATCTTC	119
*NFATC1*	GCTGTTCCTTCAGCCAATCATC	GAGGTGATCTCGATTCTCGGAC	137
*TRAF6*	CCAGTTGCACATGAGACTGTTG	AGTTTCCATTTTGGCAGTCAGC	193
*NF-KB*	AATTTGCAACTATGTGGGGCCT	ATGTCCTTGGGTCCTGCTGTTA	141
*GAPDH*	GAAGGTCGGAGTCAACGGATTT	GCCATGGGTGGAATCATATTGG	151

## Data Availability

The figure data used to support the findings of this study are included within the article.
